# Testicular Cancer Education—Hidden Potential Ways to Improve Awareness and Early Diagnosis in Young Men?

**DOI:** 10.3390/children12101380

**Published:** 2025-10-13

**Authors:** Marc Kidess, Jan Goedeke, Franz Aschl, Nikolaos Pyrgidis, Yannic Volz, Troya Georgieva, Regina Stredele, Benedikt Ebner, Michael Atzler, Darjusch Askari, Martina Heinrich, Kristina Becker, Julian Hermans, Julian Marcon, Maria Apfelbeck, Oliver Muensterer, Christian G. Stief, Michael Chaloupka

**Affiliations:** 1 Department of Urology, University Hospital of Munich, Marchioninistr. 15, D-81377 Munich, Germany; 2 Department of Pediatric Surgery, Dr. von Hauner Children’s Hospital, University Hospital of Munich, Lindwurmstr. 4, D-80377 Munich, Germany; jan.goedeke@med.uni-muenchen.de (J.G.); oliver.muensterer@med.uni-muenchen.de (O.M.); 3 School of Computation, Information and Technology, Technical University of Munich, Arcisstr. 21, D-80333 Munich, Germany

**Keywords:** testicular cancer, pediatrics, pediatric surgery, screening, well-child visits, awareness

## Abstract

**Introduction:** Testicular cancer is the most common cancer in young men. Studies show that general awareness among the risk group is low, and anticipatory guidance is of paramount importance for early detection. We queried pediatricians and pediatric surgeons on their perceived role and their interaction with patients regarding education on this issue. **Materials and Methods:** A survey was sent to pediatricians and pediatric surgeons in Germany to assess the extent of genitourinary examinations, health education about testicular cancer, and instructions for testicular self-examination during well-child visits and clinic contacts. Statistics were processed using R software (Version 4.5.1). **Results:** Data from 150 participating pediatricians and 21 pediatric surgeons were analyzed. Genitourinary examinations were performed routinely by the majority of participants, especially those in solo or group practices (*p* < 0.05). In particular, physicians who provide health education about testicular cancer perform testicular examinations significantly more often than those who do not provide such education (*p* < 0.05). Four percent of the participants offered a special consultation for male adolescents to provide information about male sexual diseases. There was a significant correlation between the length of experience of physicians and the level of health education (*p* < 0.01). **Discussion:** Although the majority of participants perform regular genitourinary examinations, only a minority provide special health education about testicular cancer or provide instructions for testicular self-examination. Most participating pediatricians and pediatric surgeons asked for more support regarding testicular cancer screening and health education.

## 1. Introduction

Testicular cancer is the most common cancer in young men, with peak incidence between the ages of 25 and 45 [1]. Symptoms include painless lumps, swelling, and heaviness in the testicles, as well as discomfort and infertility. It is necessary to differentiate this condition from other diagnoses, such as testicular torsion, which presents with painful swelling [2]. The incidence of testicular cancer has increased worldwide during recent decades [3]. Although testicular masses represent only 1–2% of solid tumors in children [4], there is, nonetheless, a need for adequate awareness and knowledge of the disease among adolescent and young-adult males. Unfortunately, studies have shown that only a minority of young men (5–10%) are familiar with the topic [5].

Pediatricians and pediatric surgeons are the primary healthcare providers during childhood and adolescence. The transition from pediatric to adult healthcare is an important and often underappreciated process, especially for patients with chronic diseases: up to 40% of patients feel that information is lost during transition [6]. Therefore, pediatricians and pediatric surgeons play an important role as a primary source of information, especially regarding reproductive and sexual health [7]. This implies that there is potential for pediatricians and pediatric surgeons to improve awareness of testicular cancer among young men.

Despite differences, most countries have recommendations for well-child visits and screening [8]. The World Health Organization (WHO) not only recommends two well-child visits during adolescence, but also specifically recommends that the genitourinary system be examined at every well-child visit. The WHO also states that well-child visits should promote healthy behaviors in children and their parents, and provide space to discuss other age-appropriate topics including sexual and reproductive health [9]. This suggests that providing information about testicular cancer during well-child visits may help improve men’s health in the future.

This study was conducted to assess the current practice patterns of pediatricians and pediatric surgeons regarding testicular cancer screening and education. To date, this is the first study which focuses especially on critical self-rating of healthcare professionals.

## 2. Materials and Methods

A cross-sectional survey was conducted among practicing pediatricians and pediatric surgeons in Germany. The questionnaire was distributed through the German Society for Pediatric Surgery, and also directly to pediatric healthcare facilities in Germany. The mailing list was provided by the German Association for Pediatrics. The study was conducted anonymously via the online tool *Limesurvey* from the University Hospital of the Ludwig Maximilian University of Munich (LMU). Access to the questionnaire and an introduction video was made available via a link or a QR code. The survey was conducted between June 2024 and September 2024. Participants were informed about the voluntary nature and anonymity of participating in this study.

The survey consisted of 24 questions, divided into two categories: 22 multiple-choice questions and 2 yes/no questions. The survey was a questionnaire which was interdisciplinarily designed to investigate current patient care related to testicular cancer in pediatrics and pediatric surgery. The items included age, education, and profession of participants, prevalence and work-up of testicular cancer diagnosis in daily routine, recommended self-examination examination frequency and technique, as well as education about testicular cancer and testicular self-examination (TSE) provided to children, adolescents, and their parents. The questionnaire can be found in Supplementary Materials. Frequency rates were defined as never (0%), seldom (<20%), often (21–50%), mostly (51–70%), and almost always (71–100%).

All categorical variables were presented as absolute numbers with proportions. We compared the categorical variables using the chi-squared test, except when the expected quantities were ≤ 5. In those cases, the exact binomial test or Fisher’s exact test was used. To determine differences between healthcare professionals, stratification was performed. Statistical calculations were performed using R statistical software (version 4.5.1). *p*-values <0.05 were considered statistically significant. This study was approved by the LMU ethics board (No. 24-0076 KB). According to the policy of the LMU ethics board, an informed consent statement was not necessary.

## 3. Results

### 3.1. Baseline Characteristics

Baseline characteristics of participating healthcare providers are displayed in Table 1. The majority of participants (34%) were between 45 and 54 years of age, 84% of participants were pediatricians, and 12% of participants were pediatric surgeons. Most participants were specialists and worked in multiphysician practices (37%) or children’s hospitals (28%). We found that 60% (*n* = 90) of pediatricians and 76.2% (*n* = 16) of pediatric surgeons worked in solo or multiphysician practice, and that most participants had more than 10 years of experience.

### 3.2. Testicular Cancer Diagnosis and Examinations

**Figure 1:**
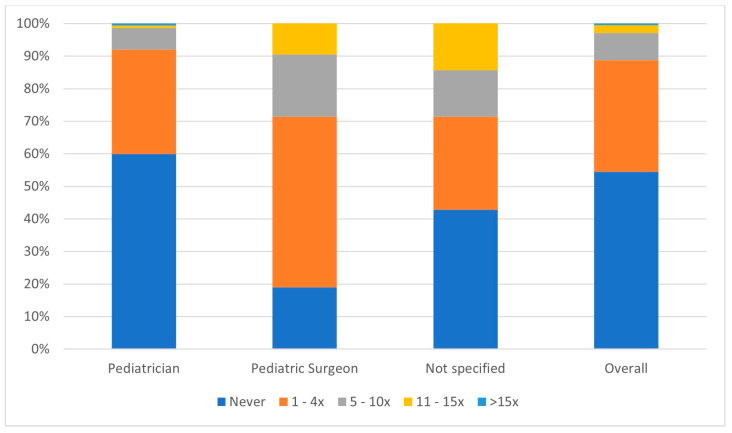
Reported numbers of times that participants had made a clinical diagnosis of testicular cancer during their career, by specialty.

The numbers of diagnoses of testicular cancer made by participants over the course of their careers can be seen in Figure 1. Most participants (55%) had never diagnosed testicular cancer during their time in practice. Pediatric surgeons reported more diagnosed cases than pediatricians.

Using Fisher’s exact test, we found that physicians in a solo or multiphysician practice were more likely to perform testicular examinations than physicians in a children’s hospital or university hospital (*p* < 0.05), although testicular cancer diagnosis was more common in university hospitals (*p* < 0.05). We did not find a significant association between the age of the physician and the frequency of testicular examinations. There was no significant association between the number of testicular cancer diagnoses made by a physician and the frequency of testicular cancer screening.

**Figure 2:**
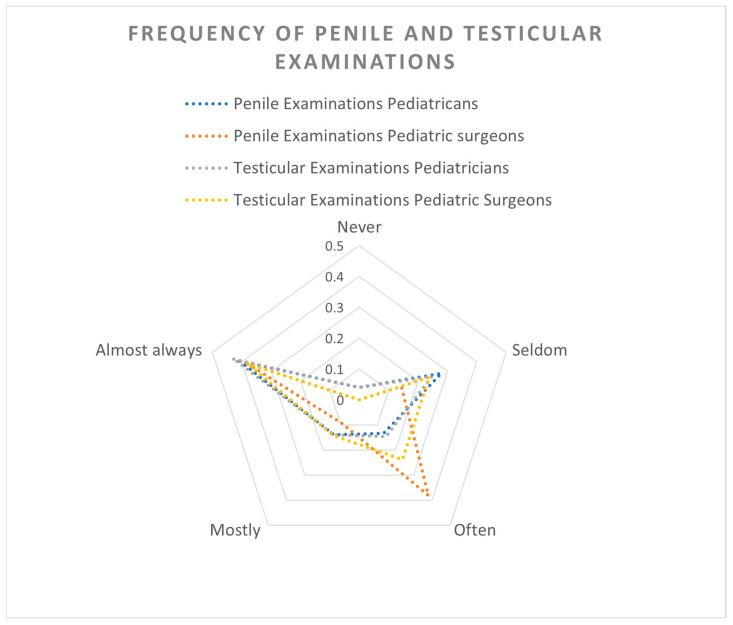
Frequency of testicular and penile examinations during visits at pediatricians and pediatric surgeons.

Figure 2 displays the frequency of penile and testicular examinations performed by pediatricians and pediatric surgeons. Overall, seven participants (3.9%) reported never having performed penile or testicular examinations. A majority reported performing penile (*n* = 69/178, 39%) and testicular (*n* = 72/172, 40%) examinations almost always during patient examinations. Fisher’s exact test demonstrated that physicians examined the testicles significantly more often than the penis (*p* < 0.01).

### 3.3. Health Education

**Figure 3:**
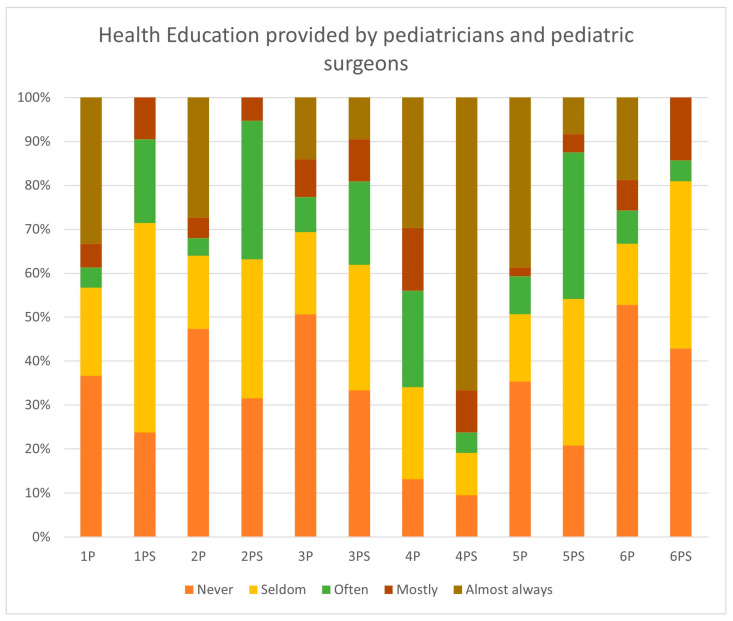
Health education provided by pediatricians and pediatric surgeons during examinations of male children. (1) Information about risk of testicular cancer and (2) age distribution. (3) Information to parents of male children regarding the risk of testicular cancer and the necessity of testicular self-examinations. (4) Information about elevated risk of testicular cancer in patients with undescended testicle. (5) Information to male children regarding the necessity of testicular self-examinations. (6) Instructions about testicular self-examinations. (P—Pediatricians, PS—Pediatric surgeons).

Figure 3 displays health education of male children and adolescents provided by pediatricians (P) and pediatric surgeons (PS).

Figure 3 illustrates data concerning frequency of providing information about testicular cancer risk, age distribution, necessity of regular testicular self-examination (TSE), and instructions on how to perform TSE during patient contacts. The highest rate (71–100% of patient contacts) of informing patients about the need for regular TSE was reported by 39% of pediatricians (*n* = 58/150) and 9.5% of pediatric surgeons (*n* = 2/21). Most pediatricians (55%; *n* = 82/150) and most pediatric surgeons (43%; *n* = 9/21) never instruct patients in TSE. A higher age of physician was significantly associated with testicular cancer education (*p* < 0.01) by Fisher’s exact test.

Overall, 3.9% (*n* = 7/178) of participants reported that they offer a special consultation for male adolescents to provide information about male sexual diseases such as testicular cancer. This service was offered by 4.8% (*n* = 1/21) of pediatric surgeons and 3.3% (*n* = 5/150) of pediatricians. Using Fisher’s exact test, we found that physicians who provided information about testicular cancer were significantly more likely to perform testicular examinations (*p* < 0.05).

In addition, 25% (*n* = 44/178) of participants reported that they have sufficient educational materials to provide their patients with relevant information about testicular cancer and testicular self-examination. The majority of participants [90% (*n* = 135/150) of the pediatricians and 86% (*n* = 18/21) of the pediatric surgeons] believed that providing more information to male patients would help increase awareness of testicular cancer and lead to earlier diagnosis of testicular cancer.

### 3.4. Knowledge of Testicular Cancer Among Male Children and Adolescents

Most of the participants rated knowledge and awareness of testicular cancer and testicular disease among male children and adolescents as low (*n* = 161/178; 90%), 6.7% (*n* = 12/178) rated such knowledge as normal, and 2.8% (*n* = 5/178) as high. Most participants (*n* = 107/178, 60%) agreed that it would be useful to provide an annual testicular check-up for male adolescents and young men covered by insurance to allow facilitate diagnosis of testicular diseases.

## 4. Discussion

This cross-sectional survey provides valuable information on the current state of anticipatory guidance and health-related education regarding testicular cancer and self-examination by pediatricians and pediatric surgeons. Our findings indicate that most pediatricians and pediatric surgeons are aware of the importance and necessity of such education, but that there are several obstacles that need to be overcome to optimize the care of male adolescents and young adults. Although most providers perform testicular and penile examinations on a regular basis, their general perception is that patients are poorly informed on the subjects of testicular cancer and testicular health. Furthermore, only a minuscule minority offers special consultations on these topics for boys and male teenagers, mostly because these special clinic visits are not adequately reimbursed by insurance companies. Another possibility for improving knowledge of teenagers and young men would be to provide them with information material. Although such material is available, it is generally addressed to adults and not teenagers.

In our study, most participants had never made a diagnosis of testicular cancer during their career. This is not surprising, given that testicular cancer usually occurs in patients in their twenties and thirties. Naturally, pediatric surgeons made more diagnoses of testicular cancer in our survey, because pediatrics is the specialty to which patients with testicular masses are referred and where they receive surgery. Furthermore, a study by Stewart et al. demonstrated that 17% of the pediatricians stopped performing GU examinations on adolescent males [10], an age group at increased risk for testicular cancer. Testicular tumors are, in fact, quite rare in children, representing only 1–2% of solid tumors in children [4]. However, this also means that pediatricians in particular should maintain a high index of suspicion when screening boys of all ages. This could help to increase the likelihood of early diagnosis.

Again, most cases of testicular cancer in children were reportedly diagnosed in children’s hospitals and children’s university hospitals (*p* < 0.05), most likely owing to the fact that such patients are referred to a hospital by a primary care provider if an anomaly of the testes is detected. It is reassuring to know that office-based physicians, whether in solo or group practice, reported a high rate of testicular examination.

A study from the United States showed that pediatricians performed a genitourinary examination in 99% of well-child visits [11]. In our study, only about half of pediatricians and pediatric surgeons performed an examination of the penis in at least 50% of their general examinations, indicating that there is much room for improvement. The WHO recommends two well-child visits during adolescence, and explicitly recommends examination of the genitourinary system at each well-child visit [9]. Unfortunately, in some countries only a minority of adolescents attend recommended well-child visits during adolescence. Although two adolescent well-care visits are generally mandated in Germany, research shows that only about 20% of adolescents at the age of 17 years attend these visits [12]. Furthermore, the older the child, the more likely the child is to visit a general practitioner, rather than a specific pediatric healthcare provider: while 95% of children under 2 years of age visit a pediatrician at least once a year, only 25% of adolescents do so [12]. This also highlights the need for other health professionals (e.g., general practitioners, internists) to participate in regular genitourinary examinations and to provide health education for young patients.

Health education for parents and children is an important pillar of well-child visits [9]. However, our study indicates that only a minority of pediatricians and pediatric surgeons regularly inform patients and parents about risks of testicular cancer, including information about age distribution, and about how to perform TSE. On a positive note, information about the risk of testicular cancer was more likely to be provided to patients with cryptorchism, who have a higher risk of developing testicular cancer. Roughly one quarter of pediatricians and three quarters of pediatric surgeons routinely informed patients with cryptorchism about the importance of testicular self-examinations in adolescence and early adulthood.

Our results show that the vast majority of participants rated the knowledge of their patients about testicular cancer as low. This may indicate a strong need for health education. Only 25% of participants reported believing that they had enough educational material about testicular cancer and TSE to provide to their patients. Most pediatricians and pediatric surgeons believe that providing more information to male patients would help to increase awareness of testicular cancer and thereby potentially lead to earlier diagnosis of testicular cancer. Studies show that little is known about testicular cancer prevention campaigns but that knowledge of such campaigns does lead to increased awareness and an increased willingness to perform TSE [13].

The WHO’s current recommendation to perform GU examinations at every well-child visit is crucial for urologic health. These examinations can support early diagnosis of testicular masses and improve patient awareness of this topic. In our study, we found that only a minority of pediatricians and pediatric surgeons regularly inform patients about testicular cancer. A Polish study investigated the reasons for delayed testicular cancer diagnoses. The researchers found that patients in a delayed diagnosis group received significantly less information about testicular cancer from healthcare professionals than patients in a timely diagnosis group (6% vs. 45%; *p* < 0.001). Furthermore, lack of knowledge also played a significant role [14]. This highlights the necessity of improving the provision of information (e.g., flyers, campaigns, special consultation hours for male adolescents) about testicular cancer to children and adolescents to improve timely diagnosis in the future.

This study has several limitations. In particular, we cannot exactly determine a recall rate, because it is unclear how many practitioners were reached. Although almost 200 pediatricians and pediatric surgeons participated, a larger number would possibly translate into a more representative sample. It is important to mention the potential for selection bias: physicians who are more interested in testicular cancer health education may have been more likely to participate in this study. In addition, this study is limited to physicians in Germany. It would be interesting to investigate differences between different European countries, practices in providing education on testicular self-examination, and specific interventions that improve compliance with guidelines. It is important to note that we were not able to objectify knowledge about testicular cancer and TSE among young patients, and we were not always able to differentiate between examinations in general and examinations during well-child visits. It is important to note that the questionnaire was not validated. Future investigations should focus on implementing validated questionnaires. Furthermore, not performing a formal a priori sample-size calculation is a limitation of this study. As an exploratory, open survey, our findings are subject to nonresponse and self-selection biases, and the precision of some subgroup estimates is limited. Future confirmatory studies should use the empirical response rates and variance estimates presented here to appropriately calculate sample sizes for specific hypotheses or effect sizes of interest.

In summary, this study is the first to evaluate the hidden potential of improving awareness of testicular cancer among young men in Germany through provision of information and health education by pediatricians and pediatric surgeons. We conclude that while pediatricians and pediatric surgeons assume low awareness of testicular cancer and testicular diseases, they have failed so far to incorporate appropriate guidance and education into their routine practice. Although a majority of participants reported performing genitourinary examinations, only a minority regularly provide health education about testicular cancer. Fortunately, we were able to show that health education for patients with risk factors for testicular cancer, such as cryptorchism, is more likely to be provided. To improve awareness among young men, health education about testicular cancer and providing instructions about TSE should be universally recommended during well-child and well-care visits. This could be achieved by implementing health campaigns that address this topic, distributing educational materials (e.g., leaflets) to patients, or using social media. One good example of a campaign that raises awareness among adolescents is the digital campaign “Be Ballsy” [15]. Studies have already demonstrated that such interventions successfully enhance patients’ awareness of the disease and intention to perform TSE [16]. As only 20% of young people attend adolescent well-care visits, it is also necessary for general practitioners and internists to provide health education especially for young men.

## 5. Conclusions

Pediatricians and pediatric surgeons play a key role in testicular cancer education. While they frequently perform examinations, they feel patient awareness is low. To address this, age-appropriate info via apps, social media, and online resources should be provided. Special male health clinics, funded by insurance, could further improve awareness and self-examination.

## Figures and Tables

**Table 1:** Baseline characteristics.

*n* = 178 (100%)	
**Sex**	
Male	*n* = 64/178 (36%)
Female	*n* = 114/178 (64%)
**Age (years)**	
<25	*n* = 0/178 (0%)
25–34	*n* = 21/178 (11.8%)
35–44	*n* = 41/178 (23%)
45–54	*n* = 60/178 (33.7%)
55–64	*n* = 50/178 (28.1%)
65–74	*n* = 5/178 (2.8%)
>74	*n* = 1/178 (0.6%)
**Specialty**	
Pediatrician	*n* = 150/178 (84.3%)
Pediatric Surgeon	*n* = 21/178 (11.8%)
Not specified	*n* = 7/178 (3.9%)
**Place of employment**	
Solo practice	*n* = 47/178 (26.4%)
Multiphysician practice	*n* = 65/178 (36.5%)
Children’s hospital	*n* = 50/178 (28.1%)
University children’s hospital	*n* = 16/178 (9%)
**Qualification**	
Trainee	*n* = 21/178 (11.8%)
Specialist	*n* = 157/178 (88.2%)
**Work experience (years)**	
<5	*n* = 20/178 (11.2%)
5–10	*n* = 20/178 (11.2%)
11–20	*n* = 53/178 (29.8%)
21–30	*n* = 49/178 (27.5%)
>30	*n* = 36/178 (20.2%)

## Data Availability

The data that support the findings of this study are available from the corresponding author upon reasonable request.

## Supplementary Materials

The following supporting information can be downloaded at: https://www.mdpi.com/article/10.3390/children12101380/s1, File S1: Questionnaire.
