# Supplementary material for: Testicular Cancer Education—Hidden Potential Ways to Improve Awareness and Early Diagnosis in Young Men?
Source: Children (Basel). 2025 Oct 13;12(10):1380. doi: 10.3390/children12101380 (PMC12562362; doi:10.3390/children12101380)
Supplement: Supplementary file 1 [file children-12-01380-s001.zip › children-3842017-supplementary.pdf]

Questionnaire

1. What is your gender?
    a. Male
    b. Female

2. How old are you?
    a. <25 years
    b. 25-34 years
    c. 35-44 years
    d. 45-54 years
    e. 55-64 years
    f. 65-74 years
    g. >75 years

3. What is your speciality?
    a. Pediarician
    b. Pediatric surgeon
    c. Others
4. What is your place of emolyment?
    a. University children's hospital
    b. Children's hospital
    c. Multiphysician practice
    d. Solo practice

5. What is your qualification?
    a. Specialist
    b. Resident

6. If you are a resident physician, what year of training are you in?
    a. 1. year
    b. 2.-4. year
    c. >4. Year
    d. I am already a specialist

7. How many years have you been working in pediatrics or pediatric surgery?
    a. <5 years
    b. 5-10 years
    c. 11-20 years
    d. 21-30 years
    e. >30 years

8. How often have you diagnosed pediatric testicular tumors or dealt with pediatric testicular tumors in your career?
    a. 1x
    b. 2x
    c. 3x
    d. 4x
    e. 5x

f. 6x
g. 7x
h. 8x
i. 9x
j. 10x
k. 11-15x
l. 16-25x
m. >25x

9. How many J1 examinations do you perform on male patients each month?
   a. <20x
   b. 20-50x
   c. 50-70x
   d. 70-100x
   e. >100x

10. How many J1 examinations do you perform on male patients each month?
    a. <20x
    b. 20-50x
    c. 50-70x
    d. 70-100x
    e. >100x

11. In what percentage of cases do you examine the penis and foreskin of boys during examinations?
    a. Never (0%)
    b. Seldom (<20%)
    c. Often (20-50%)
    d. Mostly (51-70%)
    e. Almost always (71-100%)

12. In what percentage of cases do you examine the testicles of boys during examinations?
    a. Never (0%)
    b. Seldom (<20%)
    c. Often (20-50%)
    d. Mostly (51-70%)
    e. Almost always (71-100%)

13. How often do you discuss the need for regular self-examination of the testicles with male patients during consultations each year?
    a. Never (0%)
    b. Seldom (<20%)
    c. Often (20-50%)
    d. Mostly (51-70%)
    e. Almost always (71-100%)

14. How often do you inform male patients about the risk of testicular cancer during consultations?
    a. Never (0%)

b. Seldom (<20%)
c. Often (20-50%)
d. Mostly (51-70%)
e. Almost always (71-100%)

15. How often do you inform your male patients during consultations that testicular cancer is a tumor that affects young men?
    a. Never (0%)
    b. Seldom (<20%)
    c. Often (20-50%)
    d. Mostly (51-70%)
    e. Almost always (71-100%)

16. How often do you inform patients or the parents of patients with undescended testicles about their increased risk of developing testicular cancer?
    a. Never (0%)
    b. Seldom (<20%)
    c. Often (20-50%)
    d. Mostly (51-70%)
    e. Almost always (71-100%)

17. How often do you instruct male patients to perform a self-examination of their testicles during consultations, and how often do you explain the correct procedure?
    a. Never (0%)
    b. Seldom (<20%)
    c. Often (20-50%)
    d. Mostly (51-70%)
    e. Almost always (71-100%)

18. How often do you inform the parents of a male patient about the risk of testicular cancer and the need for regular palpation examinations?
    a. Never (0%)
    b. Seldom (<20%)
    c. Often (20-50%)
    d. Mostly (51-70%)
    e. Almost always (71-100%)

19. Do you offer special consultation hours for boys, during which you provide information about preventive care and diseases of the male genitals (e.g., testicular tumors, testicular torsion, etc.)?
    a. Yes
    b. No

20. Do you have access to sufficient information material to give to patients, explaining the necessity and procedure of performing a self-examination of the testicles and providing information about testicular tumors?
    a. Yes
    b. No

21. Do you believe that providing more information (e.g., through counseling, flyers, etc.) to male patients, for example, during consultations or in general, would help to raise awareness of testicular cancer so that it can be diagnosed earlier in the future?
    a. I completely agree with this statement
    b. I somewhat agree with this statement
    c. Neutral
    d. I somewhat disagree with this statement
    e. I completely disagree with this statement

22. How would you rate awareness of testicular cancer risk among young patients?
    a. Very high
    b. High
    c. Normal
    d. Low
    e. Low

23. Do you agree with the following statement: It would be beneficial to offer an annual testicular tumor checkup or consultation for adolescents and young adults, which should be covered by statutory and private health insurance as a preventive measure in order to diagnose testicular tumors or testicular diseases in general at an earlier stage?
    a. I completely agree with the statement
    b. I somewhat agree with the statement
    c. Neutral
    d. I somewhat disagree with the statement
    e. I completely disagree with the statement

24. How would you rate the awareness and knowledge of adolescent and young adult patients regarding testicular diseases in general (e.g., testicular torsion)?
    a. Very high
    b. High
    c. Normal
    d. Low
    e. Very low
